# Predictors of latent tuberculosis infection treatment completion in the US private sector: an analysis of administrative claims data

**DOI:** 10.1186/s12889-018-5578-3

**Published:** 2018-05-29

**Authors:** Erica L. Stockbridge, Thaddeus L. Miller, Erin K. Carlson, Christine Ho

**Affiliations:** 1 Department of Health Behavior and Health Systems, University of North Texas Health Science Center School of Public Health, 3500 Camp Bowie Blvd, Fort Worth, TX 76107 USA; 2 Department of Advanced Health Analytics and Solutions, Magellan Health, Inc., 4800 N Scottsdale Rd #4400, Scottsdale, AZ 85251 USA; 3 Institute for Patient Safety, University of North Texas Health Science Center, 3500 Camp Bowie Blvd, Fort Worth, TX 76107 USA; 4 College of Nursing and Health Innovation, University of Texas at Arlington, 411 S. Nedderman Drive, Arlington, TX 76019-0407 USA; 5 Division of Tuberculosis Elimination, Centers for Disease Control and Prevention, 1600 Clifton Road, Atlanta, GA 30333 USA

**Keywords:** Latent tuberculosis infection, LTBI, Treatment completion, Claims data, Administrative data, Isoniazid, Epidemiology, Health service delivery, Public health practice, Medication adherence

## Abstract

**Background:**

Factors that affect latent tuberculosis infection (LTBI) treatment completion in the US have not been well studied beyond public health settings. This gap was highlighted by recent health insurance-related regulatory changes that are likely to increase LTBI treatment by private sector healthcare providers. We analyzed LTBI treatment completion in the private healthcare setting to facilitate planning around this important opportunity for tuberculosis (TB) control in the US.

**Methods:**

We analyzed a national sample of commercial insurance medical and pharmacy claims data for people ages 0 to 64 years who initiated daily dose isoniazid treatment between July 2011 and March 2014 and who had complete data. All individuals resided in the US. Factors associated with treatment completion were examined using multivariable generalized ordered logit models and bivariate Kruskal-Wallis tests or Spearman correlations.

**Results:**

We identified 1072 individuals with complete data who initiated isoniazid LTBI treatment. Treatment completion was significantly associated with less restrictive health insurance, age < 15 years, patient location, use of interferon-gamma release assays, non-poverty, HIV diagnosis, immunosuppressive drug therapy, and higher cumulative counts of clinical risk factors.

**Conclusions:**

Private sector healthcare claims data provide insights into LTBI treatment completion patterns and patient/provider behaviors. Such information is critical to understanding the opportunities and limitations of private healthcare in the US to support treatment completion as this sector’s role in protecting against and eliminating TB grows.

**Electronic supplementary material:**

The online version of this article (10.1186/s12889-018-5578-3) contains supplementary material, which is available to authorized users.

## Background

Up to 13 million people in the US have latent tuberculosis infection (LTBI) [1, 2]. These people are infected with *Mycobacterium tuberculosis* yet do not have active tuberculosis (TB) disease; they are asymptomatic and cannot transmit TB. Without treatment 5–10% of people with LTBI will develop TB over time, with higher progression risk among immunocompromised persons [3]. Although LTBI treatment does not eliminate the risk of progression to active TB, completion of a proven LTBI treatment regimen (e.g., 6 or 9 months of daily isoniazid, 4 months of daily rifampin, 12 doses of weekly isoniazid and rifapentine) dramatically decreases that risk [4]. The US’ strategic plan to eliminate domestic TB includes risk-targeted identification and treatment of people with LTBI [5]. This strategy is supported by the US Preventive Services Task Force’s (USPSTF) recent “Grade B” rating for LTBI testing in high-risk populations, which indicates to primary care providers that targeted LTBI testing and treatment afford moderate health benefit with little risk [6, 7].

Public health agencies have traditionally provided most TB control and prevention services in the US [8–11]. However, the USPSTF’s rating and current policy will likely drive increased involvement by private sector providers as health insurers are now required to cover TB/LTBI testing in high-risk populations with no patient cost sharing [12]. At the same time, the uninsured rate in the US is decreasing [13] and health insurance coverage is associated with increased use of primary and other private sector health care [14]. These shifts present an opportunity to coordinate public/private approaches to TB prevention. Factors associated with LTBI treatment completion are seldom studied outside of public health settings [15, 16]. Differences in patient risks, provider and patient incentives, and care processes in the private sector suggest a need for more information about the factors associated with treatment completion in this increasingly important arena.

We used a national sample of commercial claims data to examine private sector LTBI treatment across the US as a step toward filling this gap. Insurance claims offer a window into private healthcare practice patterns [17]. We aimed to use these data to identify factors associated with the completion of daily dose isoniazid LTBI treatment in the private sector setting.

## Methods

### Data source and analytic sample

We analyzed de-identified medical and pharmacy claims from Optum Clinformatics® Data Mart (formerly called the National Research Database) which includes claims for approximately 30.6 million commercially insured individuals – about 19% of the commercially insured US population [18]. We analyzed data for a randomly selected sample of 4 million people who were ages 0 to 64 years. Additional details about this sample are described elsewhere [19]. We used a claims-based algorithm to identify individual 6 to 9 month daily dose isoniazid courses of treatment for LTBI [19], which have been the most commonly used LTBI treatment regimens [20]. We examined treatment initiated between July 2011 and March 2014. In addition to requiring that data be available to determine if treatment was completed (as specified in the algorithm) [19] we required non-missing socio-demographic variables (i.e., the percent of foreign-born in county, patient location category, percent of households in county living under the federal poverty level (FPL), and state TB rate).

### Measures

#### Outcome variable

The outcome of interest was completion of daily isoniazid treatment for LTBI [21]. Patients may be prescribed a 6 or 9-month isoniazid regimen [4]. While our data do not indicate whether the 6 or 9-month regimen was prescribed, we could determine how many doses of isoniazid were dispensed. Thus, we grouped isoniazid treatments into three mutually exclusive ordinal categories: 1) non-completion (< 180 doses received within 9-months), 2) completion of the 6-month regimen but not the 9-month regimen (180 to 269 doses received within 9-months), or 3) completion of the 9-month regimen (≥ 270 doses received within 12-months) [20]. These increasing levels of completion are important because, while isoniazid treatment completion at any duration does not necessarily imply LTBI cure, the risk of progression to active TB decreases as the duration of isoniazid treatment increases [22].

#### Explanatory variables

Explanatory variables were constructed from the medical and pharmacy claims data (see Additional file 1 for details). Socio-demographic variables included sex, age, census region, and a patient location variable based on the National Center for Health Statistics urban-rural classification [23]. The percentage of households living under the federal poverty level in a patient’s county served as a proxy for household income [24]. Additional variables included insurance type (health maintenance organization [HMO], point of service [POS], or preferred provider organization [PPO]), prescription size (the supply of isoniazid received when the first prescription was filled; < 2 months or ≥ 2 months), year, and the type of LTBI diagnostic test received in the 6 months before treatment initiation. Non-clinical variables related to risk of LTBI or progression to active TB were included, such as the state TB rate. While country of birth was unavailable, we included prevalence of foreign-born individuals in the patient’s county as a proxy [25, 26]. Clinical risk factors included diabetes, tobacco use, HIV, immunosuppressive medication use, contact with or exposure to TB, and a history of or late effects of TB [27]. A simple count of each patient’s clinical risk factors represented cumulative risk (i.e., 0, 1, or > 1 risk factor).

### Statistical analyses

We calculated the proportion of individuals in each of three categories of treatment completion (i.e., < 6 months, 6 to < 9 months, ≥9 months) and examined the bivariate relationships between the explanatory variables and completion using Kruskal-Wallis tests and Spearman correlations. We explored the adjusted association between these variables and treatment completion category using multivariable generalized ordered logit models. Variables meeting the parallel-lines assumption were constrained to have equal effects; the odds ratios for non-completion versus completing ≥6 months of treatment and those for completing < 9 months of treatment versus ≥9 months of treatment were the same. Variables violating the assumption were not constrained and consequently have different odds ratios for completion category comparisons [28]. We ran two multivariable generalized ordered logit models. In Model 1 we examined the relationship between completion and cumulative risk. Model 2 explored the relationship between completion and individual clinical risk factors.

We also ran a multivariable logit model with completion of ≥6 months of treatment as the outcome measure and all predictors from the more detailed Model 2 as explanatory variables. This logit model was used to examine the reduction of variance in the treatment completion variable attributable to each predictor, which provided insight into the importance of the variables with respect to model predictions of completing ≥6 months of treatment [29, 30].

We conducted two sets of post hoc analyses. First, in order to assess the robustness of our findings we conducted sensitivity analyses using variations on our treatment completion outcomes measure. We ran four multivariable logistic regression models to explore characteristics associated with completion of ≥5 months of treatment and compare the results to the characteristics associated with ≥6 months of treatment in Models 1 and 2. Four models were used because we had two sets of explanatory variables (see descriptions of Models 1 and 2 above), and we defined completion two ways: 1) 150 doses in 9 months, and 2) 150 doses in 8 months. We explored the data using two definitions because we identified no previous studies or clinical practice guidelines defining a time period in which 150 doses (5 months) of isoniazid would be considered completed treatment.

Second, we explored our findings related to the LTBI testing variable. We ran a frequency distribution which contained additional details about the LTBI tests received. Additionally, to clarify differences between the results in our bivariate and multivariable analyses, we conducted post hoc bivariate analyses exploring the relationship between the explanatory variables and the type of LTBI diagnostic test using chi square tests for categorical variables and ANOVAs for continuous variables.

We used Stata 14.2 for most statistical testing [31] but used IBM SPSS Modeler 17 to complete the importance analysis [32]. All statistical testing was two-sided, and significance was tested at *p* < .05.

## Results

Two (0.2%) of 1074 individuals identified with the algorithm as having initiated isoniazid LTBI treatment were excluded due to missing geographic variables. Of the remaining 1072 almost half (46.2%) completed ≥6 months of treatment. The balance (53.8%) initiated but did not complete the minimum 6-months course. Roughly equal proportions completed ≥6 but < 9 months treatment or ≥ 9 months (23.6 and 22.6% of all patients, respectively; Table 1).

**Table 1:** Completion of daily-dose isoniazid treatment for latent tuberculosis infection. *N* = 1072

Isoniazid Treatment Completion	Number	% of Total	95% Confidence Interval
Less than 6 months (Incomplete treatment)	577	53.82	50.82–56.79
At least 6 months	495	46.18	43.20–49.17
≥6 months but < 9 months	253	23.60	21.15–26.24
≥9 months	242	22.57	20.17–25.18

Tables 2 and 3 describe relationships between the explanatory variables and the likelihood of treatment completion from bivariate analyses and multivariable models, respectively. Significant unadjusted non-clinical factors associated with completion included younger age, PPO insurance, larger prescription size, and residing in a county with < 15% of households below FPL. Similarly, in the multivariable models younger people (ages 0 to 14 years) had higher adjusted odds of treatment completion than older people. Compared to people in large central metropolitan counties, those in large fringe metropolitan counties had lower adjusted odds of completing ≥6 months of treatment, although this association was not seen with completing ≥9 months of treatment. Residing in a county with ≥15% of households below FPL was significantly associated with lower adjusted odds of completion. Detailed adjusted odds ratios for the associations described above are found in Table 3.

**Table 2:** Frequency distribution of patient characteristic variables for people initiating daily-dose isoniazid treatment and the proportion of people completing treatment by each characteristic. Treatment completion was categorized as 1) less than 6 months completed, 2) at least 6 months but less than 9 months completed, and 3) 9 or more months completed

		Distribution		% Achieving Each Level of Isoniazid Treatment Completion				% Completing ≥6 Mo.	
		*N*	% or Mean of Total	< 6 Months Complete [% or Mean]	≥6 but < 9 Months Complete [% or Mean]	≥9 Months Complete [% or Mean]	*p*-value: 3 Completion Levels	≥6 Months Complete [% or Mean]	*p*-value: < 6 vs ≥6 Months Complete
Sex	Female	575	53.6%	55.8%	22.1%	22.1%	0.232	44.2%	0.158
	Male	497	46.4%	51.5%	25.4%	23.1%		48.5%	
Age Group	0–14	105	9.8%	43.8%	24.8%	31.4%	0.019	56.2%	0.064
	15–29	291	27.1%	58.8%	23.4%	17.9%		41.2%	
	30–44	321	29.9%	53.9%	25.2%	20.9%		46.1%	
	45–64	355	33.1%	52.7%	22.0%	25.3%		47.3%	
Census Region	Northeast	352	32.8%	54.8%	20.5%	24.7%	0.148	45.2%	0.151
	Midwest	174	16.2%	52.3%	25.3%	22.4%		47.7%	
	South	148	13.8%	61.5%	22.3%	16.2%		38.5%	
	West	398	37.1%	53.8%	23.6%	22.6%		46.2%	
Patient Location	Large central metro county	484	45.1%	50.0%	26.7%	23.4%	0.169	50.0%	0.066
	Large fringe metro county	413	38.5%	57.6%	19.6%	22.8%		42.4%	
	Any smaller county	175	16.3%	55.4%	24.6%	20.0%		44.6%	
% of Households Under FPL in County	< 15%	596	55.6%	51.7%	22.8%	25.5%	0.035	48.3%	0.115
	≥15%	476	44.4%	56.5%	24.6%	18.9%		43.5%	
Insurance Type	HMO	188	17.5%	62.2%	21.3%	16.5%	0.005	37.8%	0.022
	POS	742	69.2%	52.8%	25.1%	22.1%		47.2%	
	PPO	142	13.2%	47.9%	19.0%	33.1%		52.1%	
INH Days Supply Received on Date of 1st Fill	< 2 month supply	991	92.4%	54.5%	24.1%	21.4%	0.020	45.5%	0.126
	≥2 month supply	81	7.6%	45.7%	17.3%	37.0%		54.3%	
Year INH Regimen Started	2011 Q3–4	230	21.5%	58.3%	23.0%	18.7%	0.308	41.7%	0.298
	2012 Q1–4	450	42.0%	54.4%	21.8%	23.8%		45.6%	
	2013 Q1–4	346	32.3%	50.3%	26.3%	23.4%		49.7%	
	2014 Q1	46	4.3%	52.2%	23.9%	23.9%		47.8%	
State TB Rate		–		3.85	3.84	3.81	0.846	3.83	0.864
LTBI Diagnostic Test	TST	441	41.1%	53.5%	22.9%	23.6%	< 0.001	46.5%	0.005
	IGRA	219	20.4%	45.2%	23.7%	31.1%		54.8%	
	Unknown/ Other	412	38.4%	58.7%	24.3%	17.0%		41.3%	
Percent Foreign Born in County		–		19.96	20.24	20.97	0.403	20.60	0.516
Count of Clinical Risk Factors	None	662	61.8%	58.0%	22.2%	19.8%	0.011	42.0%	0.002
	1	304	28.4%	47.7%	27.0%	25.3%		52.3%	
	2 or more	106	9.9%	45.3%	22.6%	32.1%		54.7%	
Diagnosis of Contact w/ TB^a^	No diagnosis	923	86.1%	54.3%	23.8%	21.9%	0.296	45.7%	0.457
	Had diagnosis	149	13.9%	51.0%	22.2%	26.9%		49.0%	
History of TB/ Late Effects	No diagnosis	1027	95.8%	54.2%	23.1%	22.7%	0.426	45.8%	0.197
	Had diagnosis	45	4.2%	44.4%	35.6%	20.0%		55.6%	
HIV Positive	No diagnosis	1030	96.1%	54.7%	23.4%	21.9%	0.004	45.3%	0.007
	Had diagnosis	42	3.9%	33.3%	28.6%	38.1%		66.7%	
Diabetes	No diagnosis	999	93.2%	54.5%	23.5%	22.0%	0.085	45.6%	0.126
	Had diagnosis	73	6.8%	45.2%	24.7%	30.1%		54.8%	
Tobacco	No diagnosis or medication	1004	93.7%	54.2%	23.7%	22.1%	0.237	45.8%	0.366
	Had diagnosis or medication	68	6.3%	48.5%	22.1%	29.4%		51.5%	
Immuno-suppressive Medication	No medication	948	88.4%	55.1%	23.0%	21.9%	0.030	44.9%	0.025
	Had medication	124	11.6%	44.4%	28.2%	27.4%		55.6%	

^a^Based on an ICD-9-CM code of V01.1. Abbreviations: *INH* isoniazid, *FPL* federal poverty level, *TB* tuberculosis, *TST* tuberculin skin test, *IGRA* interferon-gamma release assays, *LTBI* latent tuberculosis infection, *HMO* health maintenance organization, *POS* point of service, *PPO* preferred provider organization

**Table 3:** Results of two multivariable generalized ordered logit models^a^ with partial proportional odds which examine associations between patient characteristics and the completion^b^ of daily-dose isoniazid treatment for latent tuberculosis infection (*N =* 1072)

		Model 1: Includes Count of Clinical Risk Factors				Model 2: Includes Specific Clinical Risk Factors			
Independent Variables		Adjusted Odds Ratio	95% Confidence Interval		*p*-value	Adjusted Odds Ratio	95% Confidence Interval		*p*-value
Sex	Female	1.000				1.000			
	Male	1.085	0.855	1.378	0.501	1.045	0.818	1.335	0.724
Age Group	0–14	1.000				1.000			
	15–29	0.547	0.351	0.854	0.008	0.552	0.353	0.863	0.009
	30–44	0.597	0.385	0.925	0.021	0.599	0.386	0.930	0.022
	45–64	0.584	0.370	0.920	0.020	0.574	0.362	0.909	0.018
Census Region	Northeast	1.000				1.000			
	Midwest	0.934	0.588	1.483	0.772	0.933	0.587	1.484	0.771
	South	0.716	0.466	1.102	0.129	0.692	0.449	1.069	0.097
	West	0.989	0.676	1.448	0.956	0.967	0.661	1.416	0.864
Patient Location	Neither regimen completed vs. ≥6 months completed (completed 6 or 9 month regimen)								
	Large central metro county	1.000				1.000			
	Large fringe metro county	0.600	0.414	0.868	0.007	0.592	0.408	0.858	0.006
	Any smaller county	0.767	0.495	1.189	0.235	0.776	0.500	1.203	0.256
	< 9 months completed (neither regimen or 6 month regimen completed) vs. ≥9 months completed								
	Large central metro county	1.000				1.000			
	Large fringe metro county	0.800	0.537	1.193	0.275	0.791	0.530	1.182	0.253
	Any smaller county	0.767	0.495	1.189	0.235	0.776	0.500	1.203	0.256
% of Households Under FPL in County	< 15%	1.000				1.000			
	≥15%	0.628	0.469	0.841	0.002	0.609	0.454	0.817	0.001
Insurance Type	Neither regimen completed vs. ≥6 months completed (completed 6 or 9 month regimen)								
	HMO	1.000				1.000			
	POS	1.434	0.981	2.097	0.063	1.513	1.032	2.218	0.034
	PPO	1.817	1.147	2.878	0.011	1.864	1.174	2.961	0.008
	< 9 months completed (neither regimen or 6 month regimen completed) vs. ≥9 months completed								
	HMO	1.000				1.000			
	POS	1.434	0.981	2.097	0.063	1.513	1.032	2.218	0.034
	PPO	2.840	1.745	4.622	< 0.001	2.921	1.789	4.767	< 0.001
Prescription Size	Neither regimen completed vs. ≥6 months completed (completed 6 or 9 month regimen)								
	< 2 month supply	1.000				1.000			
	≥2 month supply	1.419	0.884	2.278	0.148	1.395	0.867	2.245	0.170
	< 9 months completed (neither regimen or 6 month regimen completed) vs. ≥9 months completed								
	< 2 month supply	1.000				1.000			
	≥2 month supply	2.268	1.383	3.720	0.001	2.233	1.359	3.670	0.002
Year INH Regimen Started	2011 Q3–4	1.000				1.000			
	2012 Q1–4	1.109	0.802	1.532	0.531	1.104	0.798	1.526	0.551
	2013 Q1–4	1.268	0.906	1.774	0.167	1.261	0.901	1.766	0.177
	2014 Q1	1.333	0.720	2.468	0.361	1.333	0.718	2.473	0.363
State TB Rate		0.905	0.793	1.033	0.138	0.913	0.800	1.042	0.178
LTBI Diagnostic Test	TST	1.000				1.000			
	IGRA	1.255	0.897	1.757	0.185	1.171	0.829	1.653	0.371
	Unknown/Other	0.813	0.616	1.071	0.141	0.812	0.615	1.071	0.141
Percent Foreign Born in County		1.004	0.989	1.019	0.612	1.004	0.989	1.019	0.636
Count of Clinical Risk Factors	None	1.000							
	1	1.522	1.158	2.001	0.003	na	na	na	na
	2 or more	1.816	1.188	2.778	0.006	na	na	na	na
Diagnosis of Contact w/ TB	No diagnosis	na	na	na	na	1.000			
	Had diagnosis	na	na	na	na	1.289	0.916	1.814	0.145
History of TB/Late Effects	No diagnosis	na	na	na	na	1.000			
	Had diagnosis	na	na	na	na	1.152	0.655	2.027	0.624
HIV Positive	No diagnosis	na	na	na	na	1.000			
	Had diagnosis	na	na	na	na	2.578	1.377	4.827	0.003
Diabetes	No diagnosis or medication	na	na	na	na	1.000			
	Had diagnosis or medication	na	na	na	na	1.458	0.902	2.355	0.124
Tobacco	No diagnosis or medication	na	na	na	na	1.000			
	Had diagnosis or medication	na	na	na	na	1.254	0.766	2.052	0.368
Immuno-suppressive Medications	No medication	na	na	na	na	1.000			
	Had medication	na	na	na	na	1.470	0.997	2.167	0.052

^a^Constraints for parallel lines were applied to all independent variables except patient location, insurance type, and isoniazid days supply received

^b^For both models, isoniazid treatment completion was categorized as 1) less than 6 months completed, 2) at least 6 months but less than 9 months completed, and 3) 9 or more months completed

Abbreviations: *INH* isoniazid, *FPL* federal poverty level, *TB* tuberculosis, *TST* tuberculin skin test, *IGRA* interferon-gamma release assays, *LTBI* latent tuberculosis infection, *HMO* health maintenance organization, *POS* point of service, *PPO* preferred provider organization

Insurance type and prescription size were also significantly associated with completion. The adjusted odds of a PPO-insured patient completing ≥6 months of treatment were 1.8 to 1.9 times that of an HMO-insured patient, and the odds of a PPO-insured patient completing ≥9 months were 2.8 to 2.9 times that of an HMO-insured patient. Larger prescription size was associated with higher adjusted odds of completing ≥9 months of treatment, although this association was not seen for completing ≥6 months of treatment.

**Fig. 1:**
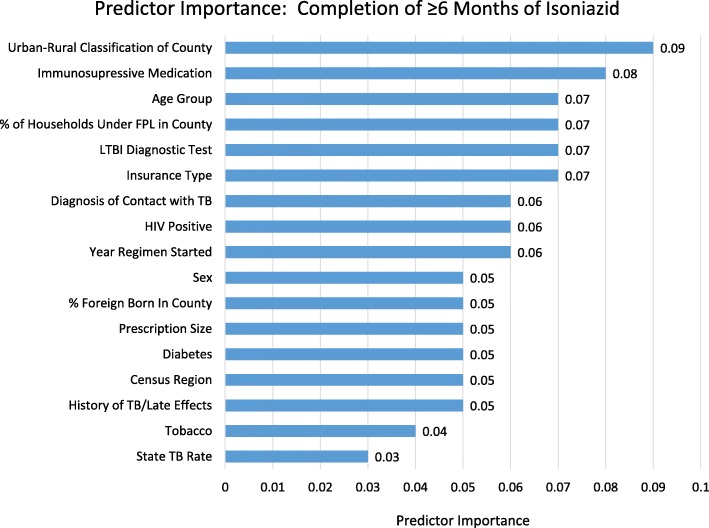
Bar chart depicting the importance of variables in predicting completion of ≥6 months of isoniazid treatment for latent tuberculosis infection (LTBI). Longer bars represent greater importance

IGRA testing, HIV, and immunosuppressive medication use each had statistically significant bivariate associations with treatment completion. In the multivariable model, people with HIV had an adjusted 2.5 times greater odds of an increased level of completion relative to those without. Additionally, both unadjusted and adjusted likelihood of completion was significantly associated with cumulative clinical risk. Compared to people with no clinical risk factors, those with one risk factor had 1.5 times greater adjusted odds and those with more than one risk factor had 1.8 times greater adjusted odds of an increased level of treatment completion. The importance analysis indicated that the most important variable in predicting treatment of ≥6 months of treatment was patient location, followed closely by immunosuppressive medication use (Fig. 1; see Additional file 2 for logistic regression model results).

The results of the sensitivity models examining ≥5 months of treatment were quite similar to the primary analyses wherein completion was defined as ≥6 months of treatment (see Additional file 3 for detailed sensitivity model results). All findings were directionally identical and odds ratios were of similar magnitude. While most variables were consistent in terms of statistical significance, there were two exceptions. Some age group and insurance type categories that were significant in the primary analyses were not significant in the sensitivity analyses. However, the *p*-values for these categories approached significance, ranging from *p* = 0.052 to *p* = 0.072. Based on these results we concluded that the results of our primary analyses were robust to variations in the definition of treatment completion.

Additional post hoc analyses indicated that 34.9% of the individuals initiating LTBI treatment had no procedure or diagnostic code in the medical claims data specifically indicating that an LTBI test occurred, although the majority of these individuals had a diagnosis of LTBI (Table 4). We also identified significant associations between LTBI diagnostic test type and our model’s explanatory variables (Table 5). Diagnostic test type was significantly associated with age, region, patient location, insurance plan type, year, clinical risk factor count, history of or late effects of TB, HIV, diabetes, tobacco use, and immunosuppressive medication use.

**Table 4:** Frequency distribution of evidence of latent tuberculosis infection (LTBI) testing occurring in the 6 months prior to LTBI treatment initiation with isoniazid (*n =* 1072)

Broad Categorization Used in Statistical Models	*N*	%	95% Confidence Interval		Detailed Categorization	*N*	%	95% Confidence Interval	
TST	441	41.1%	38.2%	41.1%	TST procedure code only, or TST code temporally first	441	41.1%	38.2%	44.1%
IGRA	219	20.4%	18.1%	23.0%	IGRA procedure code only, or IGRA code temporally first	219	20.4%	18.1%	23.0%
Other/Unknown	412	38.4%	35.6%	41.4%	IGRA & TST procedure codes present on same day	2	0.2%	0.0%	0.7%
					Other test for MTB occurred based on procedure code (no TST or IGRA code)	5	0.5%	0.2%	1.1%
					No procedure code provided information about testing, but a diagnosis code indicated that screening occurred	31	2.9%	2.0%	4.1%
					No procedure code or diagnosis code regarding testing was present, but an LTBI diagnosis code was present	261	24.4%	21.9%	27.0%
					Neither LTBI testing procedure nor diagnosis information regarding LTBI was present	113	10.5%	8.8%	12.3%

**Table 5:** Bivariate associations between *Mycobacterium tuberculosis* test type and other patient characteristics. Includes people initiating daily-dose isoniazid treatment (*N =* 1072)

		*Mycobacterium tuberculosis* Test Type			
		Tuberculin Skin Test [% or Mean]	Interferon-Gamma Release Assay [% or Mean]	Other/ Unknown Test [% or Mean]	*p*-value
Sex	Female	42.1%	19.8%	38.1%	0.767
	Male	40.0%	21.1%	38.8%	
Age Group	0–14	75.2%	8.6%	16.1%	< 0.001
	15–29	51.5%	11.0%	37.5%	
	30–44	36.1%	20.9%	43.0%	
	45–64	27.0%	31.3%	41.7%	
Census Region	Northeast	46.6%	12.8%	40.6%	0.001
	Midwest	36.8%	21.3%	41.9%	
	South	41.9%	21.6%	36.5%	
	West	37.9%	26.4%	35.7%	
Patient Location	Large central metro county	41.1%	23.4%	35.5%	0.033
	Large fringe metro county	44.1%	17.2%	38.7%	
	Any smaller county	34.3%	20.0%	45.7%	
% of Households Under FPL in County	< 15%	41.9%	20.8%	37.3%	0.672
	≥15%	40.1%	20.0%	39.9%	
Insurance Type	HMO	38.8%	13.3%	47.9%	0.015
	POS	41.1%	22.5%	36.4%	
	PPO	44.4%	19.0%	36.6%	
Prescription Size	< 2 month supply	41.5%	20.0%	38.5%	0.428
	≥2 month supply	37.0%	25.9%	37.0%	
Year INH Regimen Started	2011 Q3–4	49.1%	23.2%	38.7%	0.001
	2012 Q1–4	36.2%	21.8%	42.0%	
	2013 Q1–4	40.5%	24.9%	34.7%	
	2014 Q1	54.4%	15.2%	30.4%	
State TB Rate		3.9	3.9	3.8	0.363
Percent Foreign Born in County		21.1	20.5	19.2	0.058
Count of Clinical Risk Factors	None	46.8%	14.5%	38.7%	< 0.001
	1	36.8%	26.0%	37.2%	
	2 or more	17.9%	41.5%	40.6%	
Diagnosis of Contact w/ TB	No diagnosis	39.8%	20.6%	39.6%	0.058
	Had diagnosis	49.7%	19.5%	30.9%	
History of TB/Late Effects	No diagnosis	42.0%	20.2%	37.9%	0.031
	Had diagnosis	22.2%	36.7%	51.1%	
HIV	No diagnosis	42.4%	19.0%	36.5%	< 0.001
	Had diagnosis	9.5%	54.8%	35.7%	
Diabetes	No diagnosis or medication	42.3%	19.8%	37.8%	0.010
	Had diagnosis or medication	24.7%	28.8%	46.6%	
Tobacco	No diagnosis or medication	42.1%	19.6%	38.3%	0.011
	Had diagnosis or medication	26.5%	32.3%	41.2%	
Immunosuppressive Medications	No medication	43.8%	16.7%	39.6%	< 0.001
	Had medication	21.0%	49.2%	29.8%	

Abbreviations: *INH* isoniazid, *FPL* federal poverty level, *TB* tuberculosis, *LTBI* latent tuberculosis infection, *HMO* health maintenance organization, *POS* point of service, *PPO* preferred provider organization

## Discussion

We used commercial insurance claims data to identify important individual, clinical, and system factors associated with the completion of LTBI treatment with isoniazid. Most striking were significant associations between a patient’s insurance plan type and treatment completion, suggesting that benefit design is a potential means to modify patient behaviors and ultimately TB risk. HMO plans, the most tightly managed insurance design, were associated with the lowest likelihood of completion; PPO plans, the least restrictive plans, were associated with the highest. Completion differences may be due to differences in access or cost sharing, as such health plan characteristics are associated with continued adherence to other types of medications [32].

The lower completion rates for HMO-insured individuals suggest a need for HMOs to monitor and conduct quality improvement initiatives that improve enrollees’ LTBI treatment completion rates. Such activities would not be unusual – HMOs in most states are required to operate quality assurance programs that involve monitoring and conducting activities to improve care processes and clinical outcomes, such as improving medication adherence rates [33]. As private sector LTBI treatment becomes more common, the National Committee for Quality Assurance (NCQA) should consider incorporating an LTBI treatment completion measure into its standard set of quality performance measures (Healthcare Effectiveness Data and Information Set [HEDIS]) [34]. Health plans’ quality improvement activities often focus on improving HEDIS rates, as many states consider quality assurance requirements met if plans maintain NCQA accreditation [33] and plans are required to calculate HEDIS measures to attain and maintain accreditation [35].

Pharmacy benefit design and prescribing offer similar opportunities to decrease TB risk through improved treatment completion. Individuals filling larger prescriptions (≥ 2 months supply) had greater odds of completing a 9-month regimen. Although we cannot be certain given data limitations, completion of the longer regimen may be due to the use of mail order pharmacies with automatic refill programs. Many insurers disallow community pharmacies from providing a > 1-month supply of a medication. However, enrollees may be able to use mail order pharmacies to receive up to a 90-day supply [36], and mail order pharmacies are more likely to have automatic refill programs [37]. These programs address patient passivity and transportation barriers by mailing prescription refills at regular intervals. Thus, encouraging patients to fill larger prescriptions and use automated mail order programs may increase 9-month isoniazid completion rates so long as appropriate clinical monitoring to avoid hepatotoxicity and other complications is ensured [21].

Our analysis suggests that private sector providers are likely sensitive to and communicating the importance of treatment completion for LTBI patients at high risk of active TB. Patients with serious known risk factors such as HIV and immunosuppressive medication use [27] are more likely to complete treatment than others, and immunosuppressive medication use is of particular importance in predicting adherence. Correspondingly, completion was increasingly likely as the total number of clinical risk factors increased. Nevertheless, there are opportunities to improve completion in high-risk private sector patients, as nearly half of those with one clinical risk factor and 45.3% of those with > 1 risk factor did not complete at least 6 months of LTBI treatment. As shorter-course regimens (e.g., 3 months of weekly isoniazid and rifapentine; 4 months of daily rifampin) typically have higher completion rates [38, 39], the use of these regimens would likely increase treatment completion rates. We also found that TST is much more likely to be used among young children than IGRA. This is consistent with the CDC guidelines [40] and suggests that private providers are receiving CDC messaging related to best practices [21] and are following these practices.

We found that likelihood of completing ≥6 months of treatment varied by patient location, with individuals in large fringe metro counties (i.e., suburban counties [23]) having a lower likelihood of completion than those in large central metro counties (i.e., counties containing an inner-city [23]). These findings are in contrast to recent research examining chronic condition medication adherence for rural, suburban, and urban populations in which no significant differences were found [41]. The differing LTBI treatment completion rates that we identified may be due to differences in provider familiarity with LTBI treatment best practices. Increased provider awareness of best practices and more years of experience are associated with increasing provider adherence to best practices [42, 43]. As TB incidence is much higher in urban areas than other areas [44], providers in urban areas have likely had more exposure to patients in need of LTBI treatment, more exposure to LTBI treatment guidelines, and a greater awareness of the benefits of LTBI treatment completion. Claims data do not allow us to investigate providers’ knowledge of LTBI treatment best practices, so additional research is warranted to confirm the cause of the location-related differences. Even so, given the suburbanization of the US population [45] and the importance of this variable in identifying patients likely to complete < 6 months of treatment (see Fig. 1), our findings identify an important opportunity to improve LTBI treatment completion rates in patients treated by private sector providers in suburban areas.

Our finding that IGRA is associated with greater likelihood of treatment completion aligns with anecdotal reports that IGRA testing may yield greater diagnostic confidence for patients and providers relative to TST. However, the association is only significant in our unadjusted analysis. LTBI test type is also associated with many other variables, including clinical risk factors, census region, insurance plan type, and year. After adjusting for these other variables, there is no significant association between the receipt of an IGRA and treatment completion. It is unclear if the use of IGRA facilitates completion or if IGRA testing is more common in patients with other characteristics associated with completion.

Claims are a rich source of information about commercial insurance-covered LTBI treatment occurring across the US, but they have limitations. These data generally accurately reflect diagnoses and treatment [17], but accuracy varies with the clarity of coding instructions and guidelines [46]. There is ambiguity in the diagnostic and procedure coding for LTBI. For example, providers may be using the “contact with or exposure to tuberculosis” diagnosis code to represent LTBI status rather than known recent contacts. This might explain inconsistencies between our findings and prior reports of better completion rates among TB contacts [47–50]. Conversely, many of our findings regarding LTBI treatment completion are consistent with past research, including associations with younger age and higher income [15, 16]. Additionally, claims data only reflect information submitted to a third party payer for the purposes of reimbursement [17]. Our finding that LTBI testing procedure codes were not present in the claims for over a third of the individuals initiating isoniazid treatment suggests that some providers are either not billing for LTBI testing or some patients are receiving LTBI testing and treatment in different settings. For example, a patient might be diagnosed for LTBI in a workplace, school, or public health department that does not bill third party payers but subsequently seek treatment or fill prescriptions in the private sector using insurance benefits.

Due to limitations of claims data we cannot precisely determine treatment intent or adherence, and conclusions about provider and patient behavior are based on inference, not direct report. For instance, it is unclear whether a 6 or 9-month treatment regimen was prescribed for a given patient. Further, we cannot know if a filled prescription is actually consumed, and it is possible that those enrolled in automatic refill programs may receive refills even if they have discontinued their treatment. Of course, the uncertainty related to medication consumption applies to all medication adherence research not involving direct observation [51]. Fortunately, numerous studies have illustrated that medication adherence as measured by filled prescriptions is significantly correlated with both medication consumption and drug serum levels [52]. Consequently, claims-based methods of evaluating medication adherence are widely used in health services research and quality assurance monitoring [53–62].

Data limitations left us unable to identify important TB risk factors. Patient-level income and country of birth were unavailable. While 59% of foreign-born people in the US have private health insurance [13], claims data do not identify nativity. However, county-level nativity and FPL rates were included as proxies. Our data also did not detail treatment-related out-of-pocket costs for isoniazid or office visits, nor did it provide insight into insurance benefit plan design or network adequacy. Our analysis examining the importance of the variables in the model should be interpreted with these limitations in mind, as the results only assess the relative importance of variables available within the administrative claims data. Other, unavailable variables may be of great importance in predicting treatment completion. Nevertheless, claims data provide unique opportunities to better understand LTBI treatment occurring in a setting of increasing importance for TB prevention in the US.

## Conclusions

In the US, patient risks, provider and patient incentives or barriers, benefits design, and care processes in private healthcare differ substantially from that of public health programs. Our findings illustrate that many of these factors have an impact on LTBI treatment completion. This new information enables the development of evidence-based LTBI private sector treatment strategies. Such work is critical as more private healthcare providers provide LTBI treatment and as public health authorities consider the opportunities and limitations of private healthcare as a partner to US TB elimination efforts.

## Additional files

Additional file 1: Excel file detailing the billing codes used in the analyses. Each tab provides information about a different variable. (XLS 385 kb)

Additional file 2: Results of a logistic regression model which examines associations between patient characteristics and the completion of ≥6 months of daily-dose isoniazid treatment for latent tuberculosis infection (*N* = 1072). (XLSX 11 kb)

Additional file 3: Results of logistic regression models which examine associations between patient characteristics and the completion of at least 5 months of daily-dose isoniazid treatment for latent tuberculosis infection (*N* = 1072). (XLS 31 kb)

## Abbreviations

CDC: Centers for Disease Control and Prevention
FPL: Federal poverty level
HEDIS: Healthcare Effectiveness Data and Information Set
HIV: Human immunodeficiency virus
HMO: Health maintenance organization
IGRA: Interferon-gamma release assays
INH: Isoniazid
LTBI: Latent tuberculosis infection
NCQA: National Committee for Quality Assurance
POS: Point of service
PPO: Preferred provider organization
TB: Tuberculosis
TST: Tuberculin skin test
US: United States
USPSTF: United States Preventive Services Task Force
